# Microbial Metabolite Effects on Vasculogenic Mimicry in Metastatic Cancers

**DOI:** 10.3390/cells14110811

**Published:** 2025-05-30

**Authors:** Mohammad Kamalabadi Farahani, Aisa Bahar, Hamed Tahmasebi, Valentyn Oksenych, Mojdeh Jahantigh

**Affiliations:** 1 Tissue Engineering, Department of Tissue Engineering, School of Medicine, Shahroud University of Medical Sciences, Shahroud 36147-73943, Iran; 2 School of Medicine, Shahroud University of Medical Sciences, Shahroud 36147-73943, Iran; 3 Biochemistry Department, Faculty of Medicine, Iran University of Medical Sciences, Tehran 14496-14535, Iran; 4 Faculty of Medicine, University of Bergen, 5020 Bergen, Norway; 5 Cellular and Molecular Research Center, Research Institute of Cellular and Molecular Sciences in Infectious Diseases, Zahedan University of Medical Sciences, Zahedan 98167-43463, Iran

**Keywords:** vasculogenic mimicry, metastatic cancers, microbial metabolite, gut microbiota

## Abstract

Aggressive cancer cells can form new, functional blood vessel-like structures independently of endothelial cells, known as vasculogenic mimicry (VM), instead of the usual tumor blood vessel formation process. However, the symbiotic relationship between microbial communities and human cells ensures the upkeep of cellular metabolism and the functionality of the immune system and metastatic cancers. This interaction typically happens through the generation and management of hormonal intermediates, metabolites, secondary metabolites, proteins, and toxins. A disturbance in the balance between the host and microbiota can alter the dynamics of their relationship, creating a conducive environment for the development of diseases, such as cancers. This review aims to synthesize the initial evidence on the molecular processes governing the interactions between GM and cancer development and emphasize microbial metabolites’ effects on vasculogenic mimicry. Some microbial metabolites could also contribute to developing interactions between microbes and the tumor microenvironment. While numerous obstacles persist, GM’s immense significance and complete capability in shaping tailored anticancer plans cannot be exaggerated, highlighting the need to investigate a holistic method that includes microbial modulation therapy in cancer management.

## 1. Introduction

When cancer cells migrate to different areas of the body from where the primary tumor originated, it is known as metastasis. Despite its critical role in treatment failure and mortality, this process is still not fully understood. The most significant cause of death in cancer cases is attributed to metastasis, the characteristic feature of the disease. Malignant tumors need new blood vessel formation for their progression, infiltration, and spread [1,2,3].

The growth of tumors depends on the stimulation and growth of blood vessel cells, breakdown of the supporting tissue layer, and elongation of blood vessel cells in a linear form. Moreover, tumor angiogenesis is controlled by various vascular growth factors and factors that impede vascular growth. Complex molecular pathways are responsible for tumor metastasis and angiogenesis [3,4]. It is crucial to develop a method for identifying mechanisms that enhance cancer spread and the movement of cells to create effective targeted treatments. The process of tumor angiogenesis is not the only means of nourishing the tumor. A newly discovered mechanism known as vasculogenic mimicry (VM) has been identified as a major facilitator of metastasis and tumorigenesis in cancer, alongside sprouting angiogenesis in aggressive neoplasms [5,6,7].

Aggressive malignant tumors can create their blood supply by forming vascular channels known as VM. This phenomenon is observed in various types of invasive cancers, including breast cancer. VM, unlike traditional tumor blood vessel formation, consists of channels made of tumor cells that carry fluid and red blood cells without the involvement of endothelial vessels [6,7,8,9]. By simulating endothelial vascular function, the VM expedites tumor proliferation by ensuring efficient oxygen and nutrient supply and eliminating waste. The gut microbiota (GM), a diverse community spanning multiple kingdoms, interacts in a mutually beneficial way with the host at various body sites and is believed to play a role in the advancement, treatment, and prognosis of cancer. Since the late 1800s, the medical field has been fascinated by the connection between cancer and the microbiota, especially after William Coley’s trials with injecting bacteria to treat sarcomas, known as “Coley’s toxin” [10,11,12].

Metastatic cancers are influenced by a variety of metabolites that the GM generates. Trimethylamine N-oxide (TMAO) plays a detrimental role in the advancement of atherosclerosis and the occurrence of blood clots. In contrast, short-chain fatty acids (SCFAs) synthesized by GM contribute positively to cardiovascular well-being [4,13,14]. Recent findings indicate that the gut microbiome possesses analogous traits in its ability to produce butyrate and enhance circulating TMAO levels, thereby impacting coronary artery disease and heart failure regulation. Changes in GM and their metabolites can alleviate symptoms of metastatic cancer. Further investigation is necessary to elucidate the basic molecular mechanisms and to identify other unknown metabolites [11,12,14,15]. In the gastrointestinal tract, microbial enzymes such as choline TMA-lyase (cutC/D) and L-carnitine oxygenase (CntA/B) facilitate the conversion of dietary quaternary amines—namely phosphatidylcholine, choline, carnitine, and betaine—into trimethylamine (TMA). The liver subsequently takes TMA through the portal system, where it is converted into TMAO, chiefly by the enzyme flavin-containing monooxygenase (FMO1) present in the liver [16,17,18]. The body clears TMAO through the kidneys, which release it in urine. In conclusion, the kidneys play a crucial role in excreting TMAO through urine, thereby clearing it from the system, and various studies have shown a correlation between TMAO and VM [7,19,20].

The link between the microbial metabolite deoxycholic acid (DCA) and VM in the development of intestinal cancer remains unclear. A compelling point is that the microbiome and microbial metabolites can benefit cancer treatment by preventing cancer metastasis [10,17,21]. The microbiome’s potential to reduce inflammation might contribute to blocking the spread of cancer. Researchers are increasingly investigating the connection between the microbiome and the spread of cancer [22,23,24]. This review summarizes and puts forward the latest developments regarding the link between the microbiome and vascular mimicry in metastatic cancer.

## 2. Definition and Importance of Vasculogenic Mimicry in Metastatic Cancers

Metastatic cancer accounts for 90% of global cancer-related deaths. Understanding the mechanisms that facilitate metastasis and cell migration is essential for creating effective targeted treatments for metastatic cancer. VM is a key factor that promotes cancer metastasis and tumor development [25,26,27].

**Figure 1:**
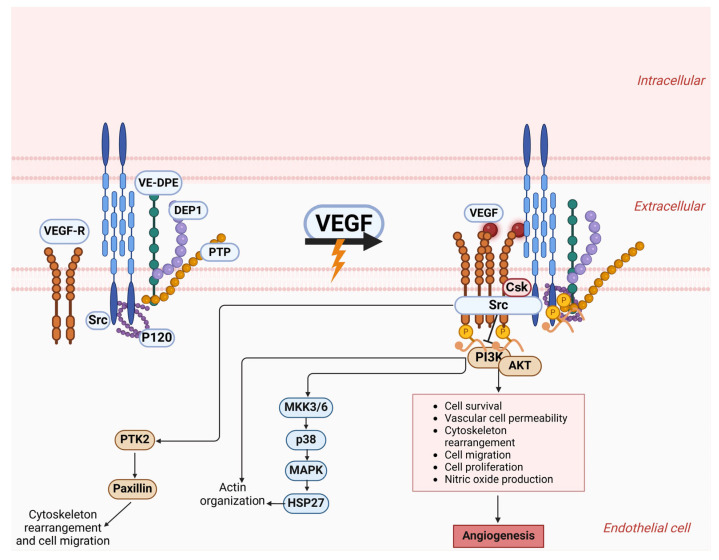
Exploration of the VE-cadherin structure and the transformations it undergoes when it comes into contact with VEGF-A. The principal component of adherens junctions is VE-cadherin, which associates with p120-catenin and β-catenin through its intracellular structure. The cytoplasmic structural domain of VE-cadherin allows it to associate with p120-catenin and β-catenin, as well as platelet hemoglobin. Also, it aids in connecting to the cytoskeletal F-actin through alpha-catenin, which leads to the assembly of the VE-cadherin–catenin complex. VE-cadherin assembles into clusters at the adherens junctions of resting endothelial cells, a state that phosphatases like VE-PTP and PTP-A help to maintain by preventing phosphorylation. The activation of VEGF-A triggers the binding of VEGF R2 with VE-Cadherin and Src, resulting in the breakdown of adherens junctions. VEGFR2 and Src kinase collaborate to phosphorylate VE-cadherin, which supports cell survival through the PI3K/AKT pathway and stimulates cell growth and migration through the ERK/MAPK pathway. Density-enhanced phosphatase-1 (DEP-1); C-terminal Src kinase (Csk). This figure was generated using BioRender software version 04.

Blood and tumor cell transportation is made easier by VM, particularly in solid tumors. Endothelial cells (ECs) are responsible for angiogenesis, whereas cancer cells are believed to perform VM. In addition to VM participating in tumor neovascularization, the clinical importance of this phenomenon lies in its potential to enhance metastasis and resistance to antiangiogenic therapy. In many tumor varieties, the presence of VM is connected with a heightened malignant phenotype [28,29,30]. Various factors from the tumor microenvironment in Figure 1, such as hypoxia, ephrin type-A receptor 2 (EPHA2), transforming growth factor -β (TGF-β), twist-related protein 1 (TWIST1), vascular endothelial growth factor (VEGF), and matrix metalloproteinases (MMPs), work together to prompt tumor cells to transition into a mesenchymal state with elevated levels of stemness markers. The clinical importance of VM and its link to tumor characteristics and clinicopathological factors make it highly significant [31,32,33,34].

## 3. Vasculogenic Mimicry: Role in the Advancement of Tumors

VM plays a role in the advancement of tumors, their spread to other parts of the body, and the presence of unfavorable clinical characteristics in cancer patients. The molecular-level analysis of genes associated with VM could offer valuable insights for developing treatment strategies for metastatic cancer, considering the significant impact of VM on cancer progression and spread [35,36]. In contrast to traditional tumor blood vessel formation, VM involves tumor cells creating fluid-carrying channels that include red blood cells without relying on endothelial vessels. The virtual model mimics the role of endothelial cells in supporting tumor growth by facilitating oxygen and nutrient delivery while removing waste efficiently [37,38,39]. As shown in Figure 1, various genes trigger VM, which is connected to tumor invasion, cancer spread, lack of oxygen, and blood vessel formation. The development of tumors through VM is strongly connected to different molecular processes, primarily including VEGFR1/2, vascular endothelial (VE)-cadherin, and MMPs. Moreover, epithelial–mesenchymal transition (EMT) is regarded as a potential process involved in forming VM and disseminating cancer [34,36,37,40].

The regulation and preservation of contact among ECs play a significant role. VE-cadherin serves as an adhesive molecule and significantly contributes to the formation of vascular structures, the control of permeability, and the process of angiogenesis in tumors. The fundamental processes of signal transmission, the intricate reconstruction of the cytoskeleton, and the integration of contractile mechanisms are all critically important for establishing and maintaining the integrity of monolayers [41,42,43]. These processes also play a vital role in the repair and regeneration of tissues, serving as the foundation for the dynamic behavior of junctions in ECs. In Figure 1, the VE-cadherin–catenin complex is the molecular foundation for adhesion junctions (AJs), which interact closely with actin filaments. Intracellular signals can be triggered, prompting endothelial cells to respond or convey alterations in junctional structures. VEGFR2 is connected to vascular endothelial protein tyrosine phosphatase (VE-PTP) through VE-cadherin and the traditional VE-cadherin–catenin complex [33,34,44,45].

If we focus on Figure 1, EC behaviors during vascular quiescence and angiogenesis are influenced by the varying activities of VE-cadherin. Proliferating cells exhibit notable alterations in the function and signaling of VE-cadherin. This transition correlates with enhanced gene transcription and the phosphorylation of proteins that possess tyrosine residues. Research indicates that endothelial cells lacking VE-cadherin exhibit a rounded shape and reduced mobility [46,47,48]. VE-cadherin molecules congregate at AJs in resting cells in the absence of vascular endothelial growth factor. As phosphatases, VE-PTP and PTP-A are essential for the maintenance of dephosphorylation processes. VEGF triggers the binding of VEGFR2 to VE-cadherin and Src, thereby interfering with the stability of adherens junctions. The interaction between VEGFR2 and SRC kinase induces tyrosine phosphorylation in VE-cadherin [49,50]. The activation of the phosphatidylinositol 3 kinase (PI3K) and protein kinase B (AKT) signaling pathways is significantly facilitated by the combination of VEGFR2 and VE-cadherin, leading to improved cell survival. The activation of the extracellular regulated protein kinase (ERK) and mitogen-activated protein kinase (MAPK) pathways is also fundamental in driving the process of cell proliferation. Moreover, the engagement of CDC42 facilitates the generation of membrane protrusions, leading to increased cellular movement [51,52,53]; Figure 1.

### 3.1. Epithelial–Mesenchymal Transition and Vasculogenic Mimicry

EMT involves transforming epithelial cells into mesenchymal cells, characterized by a reduction in cell adhesion molecules, a shift in the cytoskeletal structure from cytokeratin to vimentin, and the development of mesenchymal-like features. The role of EMT in VM formation within tumor cells is critical, as it drives their invasion and metastasis through various means. This process leads to a reduction in important epithelial markers like E-cadherin, zonula occludens-1, and α-catenin. Conversely, several mesenchymal markers show increased expression, such as VE-cadherin, fibronectin, cadherin-2, and vimentin. As a vital VM marker, VE-cadherin significantly contributes to the development of VM [46,47,54].

Important EMT-related transcription factors are Snail1, Slug (also known as Snail2), ZEB1, ZEB2, and Twist1. By binding to the E-cadherin promoter, these transcription factors modulate transcription, resulting in diminished E-cadherin levels that weaken cell adhesion and enhance invasion and metastasis. Several signaling pathways, like Notch, Wnt, and transforming growth factor-β (TGF-β), are involved in EMT. The miR34-SNAIL1 and miR200-ZEB1 pathways are major miRNA networks that epigenetically influence EMT regulation [55]. Evidence suggests that snails and slugs play a role in EMT by downregulating E-cadherin, leading to disrupted cell connections. Snai1 activation by reactive oxygen species (ROS) contributes to the advancement of cancer. It has been found that slug expression is notably connected to the cancer stem-like cells (CSCs) phenotype and VM formation in HCC. The recruitment of various chromatin enzymes to the E-cadherin promoter by ZEB1 inhibits its transcription, resulting in the loss of epithelial properties in cells. As a homolog of ZEB1, ZEB2 induces EMT. It has been found to promote tumor cell invasion and migration, raise VE-cadherin levels, and activate MMPs, contributing to vascular mimicry in liver cancer [56,57].

The action of Twist1 on E-box promoters results in EMT induction and a decline in E-cadherin levels. Reduced E-cadherin levels result in lower membrane-bound β-catenin, higher free β-catenin in the cell, and increased vimentin levels. Studies have demonstrated that twist1 is connected to VM development by regulating VE-cadherin and MMPs. These EMT-inducing transcription factors typically recognize the E-box motif in the promoters of their target genes. As a major cytokine in the tumor microenvironment, TGF-β drives the expression of numerous genes in cancer cells and is well-known for its role in regulating EMT. Some research found that TGF-β1 treatment resulted in lower E-cadherin and higher VE-cadherin levels, with ZEB2 expression rising [55,58].

Nevertheless, TGF-β had minimal effect on transcription factors such as twist1, snail1, and slug. The involvement of Wnt signaling is key to numerous physiological activities, including cell proliferation, the differentiation of endothelial cells, and angiogenesis. The induction of EMT by Wnt signaling occurs through the suppression of GSK3β, which prevents the breakdown of β-catenin. The glycosylation of Wnt ligands like Wnt1, Wnt3a, and Wnt7a occurs in the ER and Golgi, which are then delivered to the plasma membrane. During cell division, Wnts that are cell-bound can spread into surrounding tissues. Findings from prior research highlight the essential role of Wnt signaling in forming blood vessels and angiogenesis. Some research indicates that elevated Wnt3a and β-catenin levels in the nucleus are linked to the formation of vascular mimicry in colon cancer. It has been indicated that Wnt5a could facilitate VM development in epithelial ovarian cancer via the PKCalpha pathway [59,60,61,62].

### 3.2. Hypoxia and Vasculogenic Mimicry

CSCs, representing a minor fraction of the tumor, can self-renew and differentiate. Studies show that differentiated tumor cells can regain their stem cell properties and transform into cancer stem cells, with hypoxia playing a crucial role in this process. Evidence suggests that after cloning in vitro and transplanting in vivo, pluripotent melanoma cells remain viable, reproduce, and develop new tumors. CSCs can transform into different cell types; for example, glioblastoma stem-like cells grown in vitro can generate cells that exhibit endothelial traits and functions when exposed to conditions that promote endothelial differentiation [46,63,64].

CSCs are currently recognized for their role in VM development in triple-negative breast cancer and melanoma. Surface markers such as CD44, CD133, ALDH, and ATP-binding cassette transporter effectively pinpoint CSCs. There is a strong association between CSC markers and tumor invasion, metastasis, and poor outcomes. VM cells from melanoma in 3D cultures that express CD144 are also found to express the stem cell marker CD133; when this marker is silenced, the tumor’s ability to establish a VM network is significantly decreased [45,64]. How CSCs differentiate towards a pathway resembling endothelial cells is not well understood. Research in vitro demonstrated that collagen matrix pretreatment might encourage VM formation in invasive melanoma cells. However, the resulting cell count was considerably less than in vivo. When CSCs are introduced into mice alongside tumor-derived stromal cells, they tend to create more aggressive tumors, suggesting that the surrounding matrix is crucial for tumor development. These findings suggest that the microenvironment plays a crucial role in the ability of CSCs to develop into various cell types. This connection significantly shapes the evolution of VM. We assert that CSCs are positioned in a unique microenvironmental niche within tumors, identified as the CSC niche [54,65,66].

**Figure 2:**
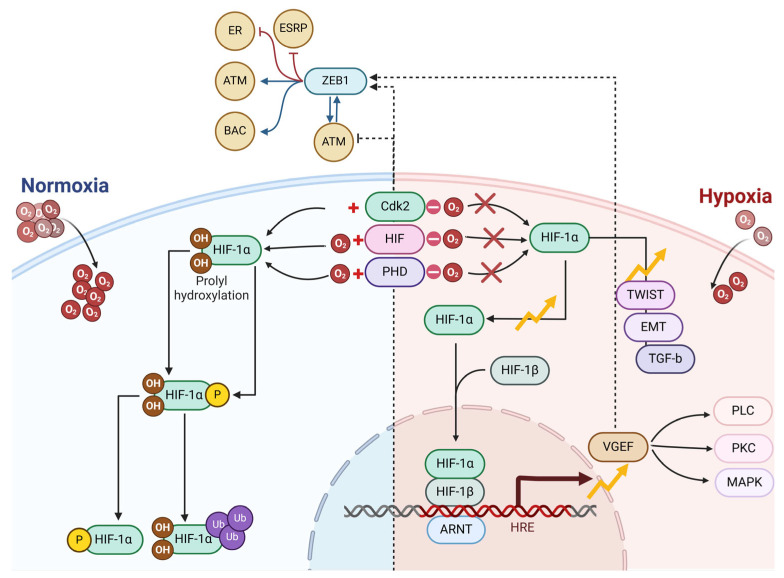
Core signaling networks that contribute to the development of vasculogenic mimicry. The signaling within vascular systems relies on VE-cadherin, EphA2, and VEGF to initiate the proteolytic cleavage of laminin 5, which results in the extracellular matrix being enriched with pro-migratory fragments γ2x and γ2’. The vascular pathway benefits from Galectin 3, as it increases the levels of VE-cadherin expression. The condition of hypoxia influences all prior pathways through its mediation of key signaling molecule expression. The Wnt proteins will likely encourage vasculogenic mimicry by engaging PKC and PI3K signaling pathways. However, they may also exhibit tumor-suppressive properties under certain conditions. This figure was generated using BioRender software version 04.

Hypoxia plays a crucial role in the CSC niche, facilitating the transformation of CSCs into structures resembling endothelial cells. The role of hypoxia and hypoxia-inducible factor (HIF) signaling pathways in cancer has been increasingly recognized, particularly in how they affect the phenotype and activity of cancer stem cells in liver, cholangiocarcinoma, colorectal, and breast cancers. It may engage transcription factors such as c-Myc, Sox-2, and Oct-4, either directly or indirectly, through pathways like adenosine/STAT3/IL-6 and MAPK/ERK to enhance the differentiation abilities of CSCs [38,66,67,68]. The presence of hypoxia-inducible factor 1 allows tumors to thrive even with limited oxygen. The activity of hypoxia-inducible factor 1 is blocked by different microbial metabolites, as illustrated in Figure 2, pointing to the potential for creating novel chemotherapy drugs based on their composition. The study focused on the connection between DCA, a microbial metabolite, and VM regarding intestinal cancer development. Findings from some research established that a high-fat dietary regimen accelerates the emergence of VM and EMT in colorectal cancer patients. The consumption of a high-fat diet led to changes in gut bacterial populations and elevated DCA concentrations in the feces of Apcmin/+ mice. Moreover, the increase in VM formation was linked to the microbial metabolite DCA, which activates the VEGFR2 signaling pathway. Moreover, the increase in VM formation was linked to the microbial metabolite DCA, which activates the VEGFR2 signaling pathway. The activation of EMT occurred alongside an increase in the expression of EMT-associated transcription factors such as ZEB1 and ZEB2, which exacerbated the process of intestinal carcinogenesis [29,48,69,70,71]; Figure 2.

Hypoxia plays a crucial role in preserving the stem-like characteristics of cancer stem cells by promoting the expression of factors associated with stem cells, including c-Myc, Sox-2, and Oct-4, while facilitating vasculogenic mimicry (VM). The transcription factors Oct4, Sox-2, Klf4, Nanog, and c-Myc are key components in stem cell biology and function as reprogramming agents, collectively called the OSKM Yamanaka factors or Y4. Elevated levels of these factors serve as functional indicators of CSCs within tumors. They can be utilized to regulate CSC activity. As a significant regulator of pluripotency, self-renewal, and maintenance, MYC can simultaneously associate with Nanog and Sox-2 by differentially binding to the HIF-2α promoter. Research consistently identifies Oct-4 and Sox-2 as the leading protein regulators that critically influence the activity of Nanog. The interaction between Klf4 and these factors results in the formation of a complex that attaches to the promoter region of Nanog, thereby facilitating its transcriptional activation. The stability of Nanog mRNA is regulated by HIF, which leads to an increase in Nanog levels in cancerous cells [45,64].

When activated in a hypoxic setting, specific pathways can indirectly influence the expression levels of pluripotent stem cells. The activation of the Notch signaling pathway by HIF-1α in hypoxic environments plays a crucial role in preserving the stem-like characteristics of cancer stem cells. γ-Secretase cleaves the Notch receptor, yielding a stable intracellular domain known as NICD. This domain is subsequently released from the membrane and translocated to the nucleus, where it interacts with CSL DNA-binding proteins, resulting in transcriptional alterations [68,69]. The Notch promoter is a direct binding site for HIF-1, which facilitates the upregulation of its downstream gene, Notch. Moreover, HIF-1α, which builds up in the cytoplasm during hypoxia, can engage directly with the internal structure of Notch. Notch undergoes nuclear translocation facilitated by its interaction with the NICD after its cleavage. The stimulation of Sox-2 promoter activity in ovarian cancer occurs directly through activating the HIF-1α and Notch signaling pathways. The transcription complex formed by HIF-1α and STAT3 targets the Vasorin gene, stabilizing Notch1 on the cell membrane, promoting NICD1 production, and inhibiting Numb’s lysosomal degradation effects [72,73].

The regulation of cancer stem cell self-renewal is significantly influenced by the Wnt/β-catenin signaling pathway. When oxygen levels are low, the Wnt signaling mechanism is initiated, resulting in the interaction between cell surface receptors (LRP-5/6 and Frizzled proteins) and the secreted Wnt ligand. When stabilized, β-catenin translocates into the nucleus, where it collaborates with the LEF/TCF transcriptional activators, leading to the binding and activation of downstream gene targets related to cancer stem cells, including c-Myc, Oct-4, Sox-2, and Nanog. The Wnt/β-catenin signaling pathway is influenced by HIF-1α, which operates in both a preceding and a subsequent manner, with these pathways exhibiting mutual regulatory effects. In low-oxygen environments, HIF-1α can promote the production of BCL9. This crucial co-activator significantly boosts the transcriptional activity driven by β-catenin [68,69,72]. The HIF-1 molecules can modulate the β-catenin co-transcription factors, LEF and TCF, in the nucleus. Likewise, HIF-2α can boost Y4 expression by indirectly activating the Wnt pathway, thereby encouraging the stem cell-like properties found in tumor cells. The heightened expression of Oct-4 and c-Myc elevates the stemness in CSCs. It stimulates VM formation, which significantly contributes to tumor invasion and metastasis. In a study on defective renal clear cell carcinoma, HIF-1α directly interferes with c-Myc’s ability to bind to its DNA partners, inhibiting its transcriptional function. HIF-2α increases the activity of c-Myc through the recruitment of binding partners or by forming a complex with them [68,72,73].

## 4. Role of Epithelial–Mesenchymal Transition and the Gut Microbiome in Vascular Mimicry

Microbial attachment to mucosal surfaces initiates EMT and breaks down the adhesion between cells in the epithelium. The interaction of bacterial adhesins with the E-cadherin/catenin complex alters cell polarity. It activates signaling pathways, facilitating EMT in epithelial cells. A study found that the onset of EMT is closely tied to immunosuppression caused by intense inflammation that impacts regulatory T cells and dendritic cells. Inflammatory cytokines are enhanced by Fusobacterium nucleatum in the colon, and Lactobacillus spp. infections in the urogenital tract lead to interleukin release [64,74].

However, EMT is vital for tumor cells that form VM, driving their invasion and metastasis by decreasing the expression of critical epithelial proteins such as E-cadherin, zonula occludens-1, and α-catenin. In contrast, specific mesenchymal markers like VE-cadherin, fibronectin, cadherin-2, and vimentin are elevated, with VE-cadherin essential for developing vascular mimicry. The transformation of epithelial cells into motile and mesenchymal forms results from extensive molecular and cellular reprogramming. The microbiota associated with cancer influences the processes of EMT and its reversal, MET, through various signaling pathways. Understanding the role of EMT/MET in cancer progression and prognosis is essential for creating new targeted therapies. Developing innovative techniques for molecular detection and bioinformatics analysis of signaling pathways impacted by dysbiosis is challenging due to the complexity of networks influenced by specific bacteria [64,74,75,76,77].

Studies suggest that the cancer-associated microbiota can encourage tumor growth and flexibility through metabolic reconfiguration. One of the main characteristics of EMT is its role in increasing resistance to anoikis, which cancer cells can accomplish by reducing mitochondrial oxidative phosphorylation; specific factors, including Snail, are crucial in managing glucose metabolism through cytochrome C oxidases. Pathogens that promote EMT typically block the adhesion of epithelial cells when they adhere to mucosal layers. Previous sections indicate that *P. gingivalis* infection in the oral mucosa increases EMT factors like Zeb1. Adhesins from bacteria attach to E-cadherin and affect cell orientation and related signaling processes. *F. nucleatum* facilitates CRC by affecting E-cadherin/β-catenin pathways through its adhesin FadA [78,79].

**Figure 3:**
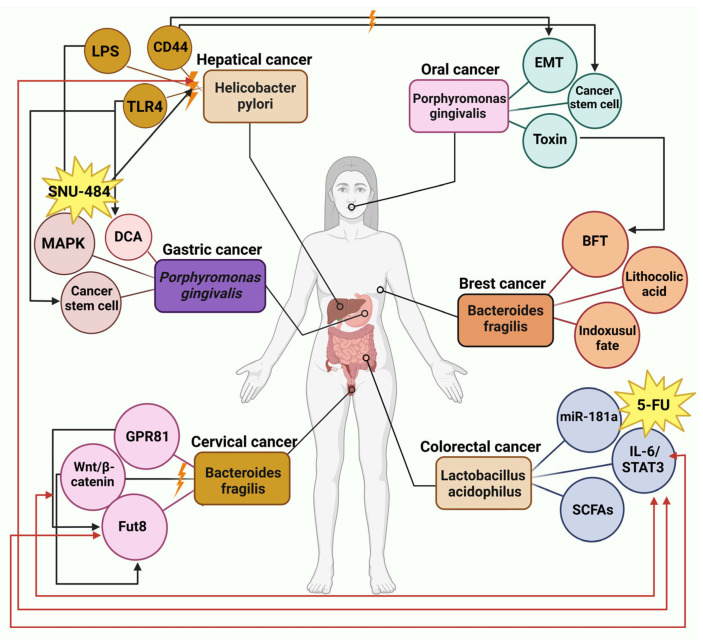
Correlations of microbiome and different cancers. Hepatic cancer: Hepatic cancer is linked to Helicobacter pylori, which is widely acknowledged as a key factor in the onset of distal gastric cancer and gastric mucosal lymphoma. Gastric cancer: Two metabolites produced by *P. gingivalis*, acetaldehyde and butyric acid, can induce cancer development. Acetaldehyde, a byproduct of ethanol metabolism, induces DNA damage and mutations while promoting the proliferation of epithelial cells. Colorectal cancer: The influence of MiR-181a-5p extends to multiple tumor attributes, encompassing cell growth, metastatic behavior, angiogenesis, the transition from epithelial to mesenchymal states, and the regulation of autophagy. Extracellular vesicles containing miR-181a-5p are discharged by colorectal cancer cells, triggering IL-6/STAT3 signaling in hepatic stellate cells. Changes in the tumor microenvironment and the formation of liver metastases occur when CRC cells and hepatic stellate cells interact. Microbes produce lactate that binds to GPR81 on cervical squamous cells, activating the Wnt/β-catenin signaling pathway. This process boosts the expression of Fut8, which encodes α-1,6 fucosyltransferase, thereby halting the progression of cervical cancer. Wnt signaling leads to the stabilization of β-catenin, facilitating its translocation into the nucleus. When nuclear β-catenin interacts with Tcf, it triggers the expression of Wnt target genes, which play a crucial role in governing stem cell behavior and tumor formation. In the absence of Wnt signaling, β-catenin undergoes continuous degradation. This figure was generated using BioRender software version 04.

For an explanation of Figure 3, among the TLRs, TLR4 has received the most thorough investigation; it detects *H. pylori* lipopolysaccharides and initiates the activation of the NF-ĸB transcription factor, which stimulates the release of pro-inflammatory cytokines. The activation of the Wnt/β-catenin pathway by CagA+ *H. pylori* in cancer cells promotes the expression of key cancer stem cell markers, which encompass CD44, Lgr5, Oct4, Nanog, and c-myc [80,81,82,83]. Following the injection of CagA into target cells, the expression of the oncogene Yes-Associated-Protein (YAP) rises significantly. When YAP is overexpressed, it causes a decline in epithelial markers like E-cadherin. It triggers EMT, thereby increasing the migratory abilities of the transfected cells [83,84,85]. In gastric cancer, the metabolites of *P. gingivalis* lead to the formation of butyric acid, which subsequently induces the generation of ROS capable of damaging double-stranded DNA or altering nucleotide structures [86]. Snai1 is activated by ROS, leading to enhanced cancer progression. Studies indicate that slug expression is closely related to the CSC phenotype and the development of VM in HCC [64].

As shown in Figure 3, extracellular vesicles containing miR-181a-5p are discharged by colorectal cancer cells, triggering IL-6/STAT3 signaling in hepatic stellate cells [71,87]. Microbes produce lactate that binds to GPR81 on cervical squamous cells, activating the Wnt/β-catenin signaling pathway [27,88]. This process boosts the expression of Fut8, which encodes α-1,6 fucosyltransferase, thereby halting the progression of cervical cancer [48,89]. The genotoxic compound colibactin, produced by *F. nucleatum* and *E. coli* strains, can attach to DNA, causing damage by stimulating the production of ROS and activating the Erk pathway. Erk activation enhances the levels of Vimentin and N-cadherin, thereby driving the occurrence of EMT. Through Toll-like receptors (TLRs), macrophages can recognize the microorganism-associated molecular patterns (MAMPs) expressed by various microbes. The production of ROS can occur in macrophages, or various signaling pathways can stimulate the release of pro-inflammatory cytokines, including IL-1, IL-6, IL-8, IL-23, and TNF. The activation of STAT3 and NF-κB signaling pathways by pro-inflammatory cytokines triggers the expression of the c-myc oncogene and MMP13, which subsequently contributes to EMT, persistent inflammation, and, ultimately, cancer development. The disruption of E-cadherin by the virulence factors FadA and BFT results in the activation of β-catenin/Wnt signaling pathways, ultimately leading to the engagement of the STAT3 and NF-κB pathways [90]. Liver cancer cells exhibited a marked elevation in the concentrations of IL-6 and phosphorylated STAT3 [91]. In both stem cells and cancer, the regulation of β-catenin is significantly influenced by the Wnt signaling pathway [90,91,92,93]. It has been shown in earlier findings that Wnt signaling significantly influences vascular growth and angiogenesis. Some studies suggest that heightened expression of Wnt3a and β-catenin in colon cancer correlates with VM formation. The Wnt5a potentially boosts VM formation in epithelial ovarian cancer through the PKCalpha mechanism [94,95].

The signaling cascade involving MAPK activation relies on the phosphorylation of various molecules upstream and downstream within the cellular environment. Signals that activate MAPK can originate from various sources, such as growth factors, hormones, cytokines, and environmental stress. These factors stimulate the phosphorylation of MAP kinase kinase kinase (also known as MAPKKK, MEKK, or MAP3K) through the action of small GTPases from the Ras and Rho families [90,96]. When ingested orally, capecitabine travels through the intestinal tract without being modified. It is first converted in the liver by carboxylesterase into 5′-deoxy-5-fluorocytidine (5′-DFCR). It is then metabolized by cytidine deaminase into 5′-DFUR in both liver and tumor tissues—the final conversion to 5-FU in tumors, facilitated by dThdPase [48,90]. In Figure 3, many forms of cancer demonstrate an aberrantly heightened activation of the IL-6/STAT3 signaling pathway, which severely undermines the immune system’s ability to combat tumors. The stimulation of the IL-6/STAT3 pathway is intricately linked to the processes of EMT and the manifestation of stem cell-like traits, which ultimately contribute to adverse outcomes in various individuals diagnosed with cancer. In oral cancer, the processes of motility and invasiveness, which EMT amplifies, are essential for the commencement of cancer metastasis. The bacteria promote increased levels of HIF-1α protein, leading to reduced E-cadherin and cytokeratin 18 while enhancing fibronectin, suggesting *E. coli*’s involvement in EMT [91,97,98].

Evidence suggests that metabolites produced by GM, such as SCFAs, are significantly associated with cardiovascular disease onset and progression. A diminished ability to produce butyrate from microbes is related to heart failure and coronary artery disease. It has been demonstrated that SCFAs disrupt the activation of endothelial cells by decreasing the synthesis of inflammatory cytokines such as IL-6 and IL-8 and by reducing the expression of adhesion molecules like ICAM-1, E-selectin, and VCAM-1 in response to stimuli like TNF-alpha and LPS. The binding of SCFAs to the G-protein-coupled receptors FFAR3 and FFAR2 seems to be linked to these processes, along with the suppression of histone deacetylases. SCFAs enhance endothelial function affected by Ang-II by blocking ROS production linked to NADPH. Butyrate and other SCFAs bolster the intestinal lining and improve the integrity of venous endothelial junctions [99,100,101].

## 5. Role of Hypoxia and the Gut Microbiome in Vascular Mimicry

A hypoxic environment can alter the host GM and produce metabolites that affect physiological levels. A study on mice exposed to hypoxia revealed altered gut flora diversity and metabolism [45,68]. Researchers examined the gut bacteria of mice exposed to varying altitudes and oxygen concentrations, using the mice as subjects. Variations in GM diversity were observed in mice fed at varying altitudes, according to the study. Changes in diversity were observed in rats within 5 days of being in or out of a hypoxic chamber, as indicated by metagenomic sequencing. Researchers have established a strong relationship between the place where an individual resides and the differences in their GM [45,69].

### 5.1. Intestinal Hypoxia and Host Microbiota

Microbial enzymes are essential for breaking down dietary fibers in the colon, as the body’s enzymes cannot process these fibers. The intestine predominantly contains acetate, propionate, and butyrate, the soluble fatty acids created and released through this process. These SCFAs act on host cells through receptor activation and inhibition of histone deacetylases, leading to various cellular effects [102]. The effects of SCFAs arise from several mechanisms, including the activation of GPRs, inhibition of HDACs, and changes in metabolism. GPR41 preferentially binds butyrate and propionate over acetate, while GPR43 has a higher affinity for acetate than the other two. GPR109a, a receptor for niacin, shows a strong attraction to butyrate and ß-hydroxybutyrate. Expressed in the kidney’s juxtaglomerular apparatus and small vessel endothelium, Olfr78 is an olfactory receptor that helps control blood pressure by modulating renin levels and facilitating peripheral vasodilation. GPRs that bind SCFAs are located in several areas of the body, especially on immune cells, but also on intestinal epithelial cells, cells of the autonomic nervous system, mammalian tissues, adipocytes, the juxtaglomerular apparatus in the kidneys, and vascular endothelium, allowing SCFA effects in various organs [1,102,103].

SCFAs are a significant energy source that increases mitochondrial oxygen consumption in Caco-2 cells, activating HIF-1 and its target genes [102,104]. This advances barrier strength, indicated by a 30% decrease in FITC-dextran permeation in butyrate-treated cells. Germ-free mice and those treated with antibiotics exhibit lower concentrations of SCFAs in the colon, disrupted physiological hypoxia, and reduced activation of HIF-1. Nevertheless, these consequences can be overturned with the oral intake of tributyrin, a pro-drug of butyrate. Impairment of the epithelial barrier is observed in T84 cells following lentiviral-induced HIF-1β knockdown, underscoring the link between butyrate and HIF-1 activation in intestinal epithelial cells [105].

The relationship between HIF-1α and the Wnt/β-catenin pathway is bidirectional, with both pathways regulating each other. HIF-1α triggers the production of BCL9 under hypoxia, which is essential for enhancing the transcriptional activity of β-catenin. HIF-1 molecules can directly influence the nuclear co-transcription factors LEF and TCF associated with β-catenin. The activation of the Wnt pathway by HIF-2α leads to increased Y4 expression, promoting stem cell-like properties in tumor cells. The heightened expression of Oct-4 and c-Myc elevates the stemness in CSCs. It stimulates VM formation, significantly contributing to tumor invasion and metastasis [94,95,106].

The health implications of the human GM are closely tied to diet, environmental exposures, and lifestyle choices, which influence its composition and metabolic functions. The ability of CSCs to maintain their stem cell properties or control their growth is closely tied to the resources provided by their surroundings. Studies have tried deciphering how CSCs respond to different diets [104]. The diverse anaerobic microbial community breaks down undigested food and substances produced by the host, especially mucin, to generate various metabolites that mirror the chemical complexity of the diet and the microbiota’s distinct way of processing them. Key components in the microbiota–cancer association are the metabolites produced through microbial metabolism, known as microbiota metabolites. The classification of metabolites depends on their source (intracellular or extracellular) and their function (primary or secondary). Essential functions like growth, reproduction, or development do not require the production of secondary metabolites during the phase of stationary growth [107,108].

### 5.2. Trimethylamine N-Oxide

**Figure 4:**
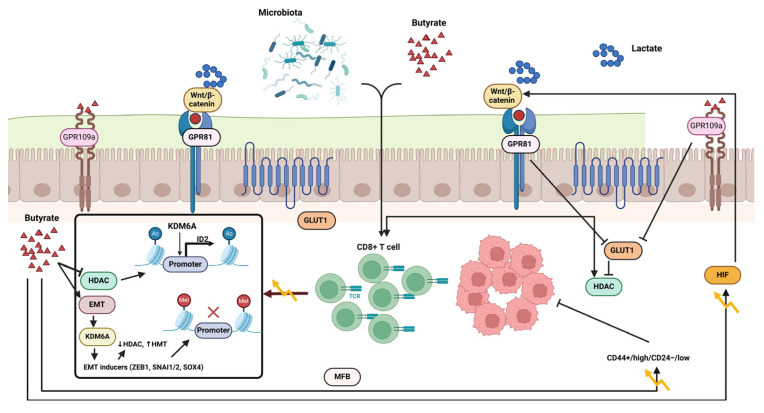
Pathways in immune cells that short-chain fatty acids generated by GM triggers. The interaction of short-chain fatty acids such as propionate, butyrate, and acetate with the host microbiome significantly affects intestinal immune responses. This interaction promotes the development of regulatory T cells (Tregs), enhances the activity of dendritic cells (DCs) and macrophages, stimulates the production of anti-inflammatory cytokines, and supports the proliferation of plasma B cells and antibody generation. These processes occur through the inhibition of histone deacetylase (HDAC) and the activation of GPR109A and GPR43, resulting in anti-inflammatory outcomes and improved tolerance to microbial threats. This figure was generated using BioRender software version 04.

The association between TMA, TMAO, and the emergence of atherosclerosis has been thoroughly explored in various studies. Figure 4 highlights the involvement of GM in the generation of TMAO. Gut microbiota, particularly from the Clostridia and Enterobacteriaceae families, synthesize TMA during the breakdown of nutrients like carnitine, choline, and lecithin, which are present in dietary sources such as meat and eggs [109]. The hepatic enzyme flavin mono-oxygenase (FMO)-3 oxidizes absorbed TMA into TMAO. TMAO plasma concentrations exhibit considerable variability, both intra-individually and inter-individually, making it difficult to draw comparisons between studies. Additionally, TMAO concentrations tend to be greater in females, which can be attributed to variations in the expression of the converting enzyme FMO3 and the higher rates of excretion found in men. The excretion of TMAO mainly occurs in the kidneys through processes such as glomerular filtration and tubular secretion, which accounts for the increased levels of TMAO associated with reduced renal function [109,110].

TMAO triggers inflammation in endothelial cells and promotes their transition to a mesenchymal state. By increasing platelet reactivity and promoting their activation and deposition, TMAO plays a role in advancing atherosclerosis. It also disrupts endothelial cell function and encourages apoptosis through heightened adhesion molecule levels. TMAO supplementation in mouse models increases macrophage cholesterol buildup and contributes to atherosclerotic plaque development. TMAO triggers the p38 MAPK/ERK/NF-κB signaling pathway in primary human aortic endothelial and vascular smooth muscle cells. This leads to increased inflammatory factor expression and a potential rise in atherosclerosis and cardiovascular disease risk. TMAO interferes with eNOS phosphorylation, diminishing nitric oxide release and causing issues with endothelial health. Consequently, vascular relaxation and formation are inhibited [111,112,113].

By boosting the production of ROS, TMAO causes oxidative stress in cellular environments [109,114]. Elevated ROS levels can cause damage to important cellular parts like DNA, proteins, and lipids. This excess of ROS can activate pro-inflammatory pathways, including the NF-κB pathway. Increased NADPH oxidase activity, linked to ROS production, is associated with TMAO. This increase can lead to higher levels of ROS and greater oxidative stress in cells. Evidence shows that TMAO enhances the levels of pro-inflammatory cytokines, such as TNF-alpha and IL-1B. At the same time, it lowers the amount of anti-inflammatory cytokines, notably IL-10. FMO3, an enzyme in the liver, is vital for lipid metabolism regulation [113,115]. The silencing of FMO3 in mice fed a diet rich in cholesterol led to a decrease in intestinal lipid uptake, a reduction in hepatic cholesterol production, and an increase in reverse cholesterol transport, collectively contributing to restoring cholesterol equilibrium. Evidence suggests that TMAO elevates platelet responsiveness, promoting thrombosis and thereby resulting in thrombotic occurrences linked to atherosclerotic conditions [114].

Tumor-bearing mice with raised TMAO levels exhibit significant tumor growth, more blood vessel formation, and increased VEGFA and CD31 concentrations. The tumor-enhancing properties of TMAO shed light on how high red meat and choline consumption relate to a greater risk of CRC. Elevated VEGF levels in malignant melanoma are connected to the formation of VM. Vascular endothelial cells, tumor cells, and additional cells in the tumor microenvironment express VEGFR1 and VEGFR2. The activation of the PI3K/AKT signaling pathway by VEGFR2 is vital for endothelial cell proliferation. PI3K triggers targets like MT1 MMP and MMP-2. Laminin 5 gamma 2 chain (LN-5γ2) is split into 5 gamma 2 prime (5γ21) and 5 gamma 2x (5γ2x) through the joint action of MT1 MMP and MMP-2. Combining these two fragments in the microenvironment ultimately results in the development of the VM network structure [116,117,118].

### 5.3. Butyrate

In Caco-2 cells, butyrate enhances mitochondrial oxygen use, triggering the activation of HIF-1 and its target genes. Butyrate treatment enhances barrier strength, as shown by a 30% reduction in FITC-dextran permeability in the cells. Germ-free and antibiotic-treated mice present lower SCFA levels in the colon, disrupted hypoxia, and reduced activation of HIF-1. However, the effects can be reversed by taking tributyrin, a precursor to butyrate. T84 cells show a compromised epithelial barrier after HIF-1β knockdown via lentiviral methods, highlighting the connection between butyrate and HIF-1 activation in intestinal epithelial cells [119,120,121].

As shown in Figure 4, resistant starch and polysaccharides, which are non-digestible carbohydrates, make their way to the colon without being broken down because specific digestive enzymes are lacking. Gut bacteria create SCFAs and gases by breaking down substances without oxygen [122,123]. Evidence suggests that a high-fiber diet is associated with a decreased risk of CRC, prompting researchers to delve into the potential of SCFA in preventing carcinogenesis. Nevertheless, butyrate treatment has been noted to inhibit the multiplication of adenocarcinoma cells while supporting their differentiation and death, with no effect on the regeneration of healthy epithelium in different experimental setups [122,124].

The activation of the G protein-coupled receptor 109a (GPR109a)–AKT signaling pathway has been proposed as a potential rationale for the butyrate paradox. The reduction of membrane G6PD and GLUT1 levels significantly hinders DNA synthesis and glucose metabolism in CRC cells. In malignant colon cells that rely on glycolytic metabolism, butyrate builds up and functions as an A histone deacetylase (HDAC) inhibitor, impeding cell cycle advancement by modifying gene expression [119,120,121].

Different metabolic processes in mature and immature colon cells could explain the butyrate paradox. Colon cells that are specialized create a metabolic barrier by utilizing butyrate to generate a butyrate gradient, which substantially inhibits the proliferation of stem cells. Colonocytes may favor the breakdown of butyrate rather than propionate and acetate for a specific reason [125,126]. A novel form of metformin called Metformin-butyrate (MFB) has shown enhanced efficacy in specifically attacking the CD44+/high/CD24−/low cancer stem cell-like group in breast cancer and blocking the growth of mammospheres in contrast to regular metformin. Butyrate was found to boost the chemotherapeutic impact of 5-FU on CRC cells, hinting at its ability to make CRC cells more responsive to chemotherapy. The presence of butyrate in organoids cultured in 3D from CRC patients significantly boosted the efficacy of radiation in inducing cell death and improving therapeutic effects compared to radiation treatment alone [127].

### 5.4. Secondary Biliary Acids

Dietary fatty acids can improve the self-renewal and tumor-initiating capabilities of intestinal stem and progenitor cells. Cholic acid and Chenodeoxycholic acid are indispensable substances that serve as signaling molecules for the efficient breakdown and absorption of fats from the diet. The liver carries out enzymatic processes that transform cholesterol into primary bile acids (BAs), which subsequently bond with either glycine or taurine [128,129]. A small fraction of bile acids are absorbed into the colon each day in humans because they are recycled in the terminal ileum through an active transport process. The GM converts primary bile acids into secondary bile acids like DOC and lithocholic acid (LCA). Approximately 30–40% of the circulating BA pool is composed of cholic acid and chenodeoxycholic acid, with DOC making up 20–30% and LCA less than 5% [130].

**Figure 5:**
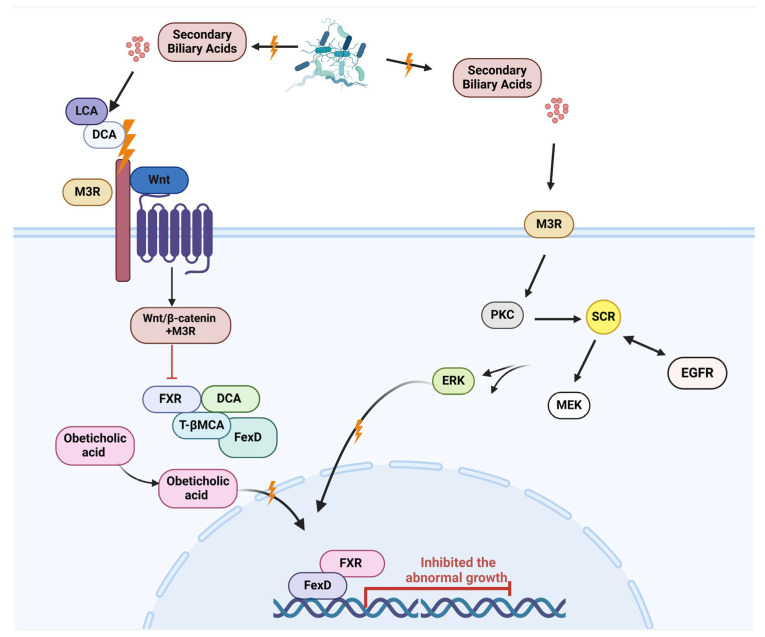
A visual representation detailing the pathways that play a role in bile acid synthesis and metabolic processes. Intestinal bacteria in the ileum catalyze the deconjugation of bile acids (BAs), converting them into secondary bile acids such as DCA and lithocholic acid (LCA). Most unconjugated bile acids undergo reabsorption in the colon. They are directed to the liver through the portal vein, allowing them to contribute to another digestive cycle called enterohepatic circulation. Heparin-binding epidermal growth factor (HB-EGF) triggers the transactivation of the epidermal growth factor receptor (EGFR) after being released from pro-HB-EGF by matrix metalloproteinase-7 (MMP-7). The activation and expression of MMP-7 are also consequences of M3R activation, creating a ‘feed-forward’ effect. The signaling process continues downstream through the ERK1/2 and PI3K pathways, causing gene expression shifts that promote cancer advancement. This figure was generated using BioRender software version 04.

In Figure 5, secondary bile acids (Bas) regulate different physiological and pathological processes, which act as signal molecules through various signaling pathways. Changes in the GM can impact the equilibrium of primary and secondary bile acid reservoirs, leading to specific pathological bile acid compositions. The interaction between GM and bile acids can lead to tumor growth by regulating immune cells, suppressing anti-tumor immune responses, and boosting Treg activity, fostering immune-suppressed environments that support tumor development [128,129]. A study identified a favorable connection between the colon-derived unconjugated fraction of DCA and the creation of colorectal adenomas, which are early indicators of CRC. Different research found that unbound secondary bile acids, specifically DCA and LCA, impact muscarinic acetylcholine receptor M3 (M3R) and Wnt/β-catenin signaling, encouraging the development of cancer stem cells in the cells lining the colon [82,131]. Furthermore, secondary biological agents can potentially promote the formation of CSCs originating from both malignant and non-malignant cells. Responsible for overseeing the negative feedback loop of bile acid synthesis in the ileum and liver, FXR (FXR) is a crucial factor in regulating the proliferation of intestinal stem cells [131,132,133].

The inhibition of intestinal FXR by DCA and tauro-β-muricholic acid (T-βMCA) has been linked to enhancing cancer cell proliferation and DNA damage. This activation can slow down the growth of the tumor. The validity of this idea was established through the administration of the FXR agonist drug Fexaramine D (FexD) [133,134]. Two bacterial strains were found in a different study that can influence intestinal FXR activation directly. In mice, FXR activation and the upregulation of FXR-dependent genes can be initiated by the cell-free supernatants from *Bacteroides dorei* and *Eubacterium limosum*. These findings suggest that these strains may have a positive impact as probiotics, especially when a high-fat diet could disrupt the bile acid pool, potentially leading to an increased risk of colorectal cancer [135,136]. According to a new study, combining a nutritious diet and medication can decrease the spread and attachment of cancer cells in secondary tumors. Research suggests that the disruption of intestinal FXR by T-β-MCA and DCA promotes the growth of Lgr5+ cells while damaging DNA. In contrast, the specific activation of FXR using Fexaramine D (FexD) and Obeticholic acid (OCA) lessened the unusual increase of Lgr5+ cells. It prevented the development of colorectal cancer (CRC) [136,137,138].

Evidence suggests that gastric cancer cells synthesize and release acetylcholine, facilitating the autocrine and paracrine activation of M3R, which subsequently enhances the proliferation of gastric cancer cells through the transactivation of the epidermal growth factor receptor (EGFR) signaling cascade. The microbiota modulates TGR5 signaling in bile acid metabolism by producing agonists. In contrast, FXR signaling is influenced by the metabolism of antagonists [81,138]. The metabolic influence of TGR5 and FXR is substantial, and an altered microbiota can affect host physiology by changing the signals processed through these receptors. For the microbiota to promote obesity, steatosis, and reduced glucose and insulin tolerance, it is essential to have the capacity to metabolize TauroMCA, which acts as a naturally occurring antagonist of FXR [139,140].

Within pancreatic beta cells, the concentrations of FXR and TGR5 are notably high, and these receptors positively impact both insulin production and the secretion of insulin that occurs in response to glucose. Activation of TGR5 within pancreatic α cells increases proconvertase-1 expression, which redirects glucagon synthesis towards GLP-1, consequently boosting the density and performance of β cells through paracrine interactions [141]. In HK2 cells under hypoxia and in mice with AKI, FXR activation diminishes the generation of reactive oxidative species. The FXR agonist OCA, when administered beforehand, prevents the rise of renal NADPH oxidase in LPS-triggered AKI. The regulation of renal NADPH oxidases by FXR effectively lowers oxidative stress, as demonstrated by the natural ligand CDCA and the synthetic ligand OCA [117,142,143].

In cancer metastasis, ROS are crucial and are believed to influence or regulate EMT. Epithelial cells undergo EMT by losing their E-cadherin markers and gaining mesenchymal markers like Snail and Vimentin. In tumor cells, Snail inhibits E-cadherin expression, and activating the PI3K/Akt pathway is vital for ROS-mediated EMT in colon cancer. This process involves ROS stimulating Akt, increasing Snail and Vimentin, and lowering E-cadherin [144,145]. ROS is crucial for regulating angiogenesis, which is important for cancer spread; it induces HIF-1α expression and stabilizes it, increasing VEGF and promoting angiogenesis. ROS enhances HIF-1α and VEGF levels during the malignant transformation of human bronchial epithelial cells, primarily driven by the ERK and PI3K/Akt pathways. Focusing on ROS could effectively lower VM levels [117,143,146].

## 6. Gut Microbiota Metabolites and Cancer Therapy

New research indicates that metabolites produced by bacteria associated with tumors can affect how well conventional cancer therapies, such as radiotherapy, immunotherapy, and chemotherapy, work. Collaborating bacterial metabolites with traditional cancer therapies is a pivotal innovation in pursuing more effective combination treatments [147,148].

### 6.1. Chemotherapy Efficacy

While chemotherapy is vital for advanced cancer treatment, a significant number of patients experience resistance, resulting in few benefiting from it. Evidence strongly supports that bacteria and their metabolites are key players in regulating chemotherapy’s pharmacokinetics, resistance, toxicity, and efficacy by influencing drug metabolism and the host’s immune response. Bacteria associated with tumors have diverse enzymatic capabilities that impact the response to chemotherapy and its adverse effects. Their metabolic reactions mediate the impact of microbial enzymes on chemotherapy effectiveness. Serving as a first-line treatment, gemcitabine, a cytidine analogue, is utilized in managing advanced triple-negative breast cancer and pancreatic ductal adenocarcinoma (PDAC) [149,150,151]. The enzyme deoxycytidine kinase catalyzes the transformation of gemcitabine into gemcitabine diphosphate and triphosphate, inhibiting DNA synthesis and potentially resulting in programmed cell death. Within PDAC tumor environments, Gammaproteobacteria can metabolize gemcitabine into the non-active form 2′, 2′-difluoro deoxyuridine through the enzymatic action of cytidine deaminase, thus reducing its efficacy against cancer. Consequently, using inhibitors that affect microbial metabolic enzymes may offer a promising avenue for cancer therapy. The administration of chemotherapy can result in significant side effects, such as diminished blood cell levels and gastrointestinal issues, potentially leading to the postponement or cessation of the scheduled treatment [149,152].

The use of chemotherapy changes the intestinal microbiota, resulting in dysbiosis. The microbiome’s structure may impact the intensity of side effects from treatment. Different methods are being introduced to reestablish microbial equilibrium following chemotherapy or radiotherapy to reduce side effects. Chemoresistance is influenced by bacteria associated with tumors. *F. nucleatum*, found in higher levels in CRC than adjacent healthy tissue, has been linked to tumor growth and adverse outcomes. Findings from a recent study suggest that elevated *F. nucleatum* levels may cause resistance to oxaliplatin and 5-FU. *F. nucleatum* influences the TLR-4/MYD88 signaling mechanism, impeding apoptosis and activating autophagy, leading to increased chemotherapy resistance. Lactobacillus plantarum supernatant aids in making CRC cells more sensitive to 5-FU [149,150,151]. Butyrate, a type of SCFA, enhances the cancer-fighting properties of gemcitabine and irinotecan by promoting cell apoptosis. By increasing IL-12 receptor levels and recruiting cytotoxic CD8+ T cells, butyrate can enhance the effectiveness of oxaliplatin against cancer. A recent investigation highlights that the microbiota-derived metabolite indole-3-acetic acid (3-IAA) is significantly present in pancreatic ductal adenocarcinoma patients who respond to chemotherapy, with serum concentrations linked to enhanced survival rates. Overall, bacterial metabolites are important in chemotherapy drugs’ toxicity, resistance, and effectiveness. This suggests that a new cancer treatment strategy could target these bacteria and their metabolites [149,153].

### 6.2. Immunotherapy Response

Over the past decade, significant progress has occurred in cancer immunotherapy, particularly regarding immune checkpoint blockade (ICB) therapy. A considerable proportion of individuals, especially those with a reduced mutational burden, face difficulties due to the inadequate effectiveness of immunotherapy. Recent findings demonstrate that the composition of GM and its byproducts play a crucial role in modulating the host’s immune responses and enhancing anticancer mechanisms [154,155]. Research involving mouse models of cancer demonstrates that the effectiveness of ipilimumab in treating tumors is reduced in both germ-free environments and when antibiotics are administered. Studies demonstrate that Bifidobacterium can potentially increase the effectiveness of immune checkpoint blockade therapy by activating dendritic cells and facilitating the movement of cytotoxic CD8+ T cells into the tumor microenvironment. Patients with metastatic melanoma who exhibit increased quantities of Faecalibacterium prausnitzii and Gemmiger formicilis tend to respond better to anti-CTLA-4 therapy [156,157].

### 6.3. Radiotherapy Efficiency

This cancer treatment, known as radiation therapy, is clinically validated for various cancers. Two essential factors underpin the principles of radiotherapy. This approach employs ionizing radiation to eradicate cancer cells by harming their DNA. By generating reactive oxygen species, RT indirectly inflicts DNA damage on cancer cells. Although RT successfully kills cancer cells, it can also adversely affect normal tissues and the beneficial microbes in the intestines. The gut microbiome analysis post-radiation therapy revealed a drop in friendly bacteria like Bifidobacterium, Faecalibacterium, and Clostridium species. Findings indicated that treating mice with vancomycin improved the effectiveness of radiotherapy by targeting bacteria responsible for SCFA production, which can be exacerbated by fungal overgrowth from broad-spectrum antibiotics [158,159].

## 7. Conclusions

In conclusion, metabolites from GM significantly influence tumor development and advancement; metabolites from GM, particularly SCFAs, BAs, TMAO, and butyrate, influence VM. By reshaping the tumor microenvironment, microbial metabolites play a role in cancer progression, affecting immune cell functions and cytokine levels. Various signaling pathways, including MAPK, PI3K/Akt, NFκB, and Wnt, are regulated by microbial metabolites, impacting the growth, apoptosis, and metastasis of cancer cells and VM. EMT plays a role in acquiring CSC properties, with EMT-inducing CSCs seen as a key source of CSCs and a target for VM formation in cancer. Focusing on EMT and microbiota may improve anti-VM treatment, leading to less invasion and metastasis and better patient survival. Inhibiting VM can restrict tumor cell movement along blood vessels, and due to the connection between epithelial–mesenchymal transition and tumor spread, it is important to create drugs that target vascular mimicry to limit invasion. The significance of microbes in health and disease is recognized. However, their role in facilitating cancer through EMT is not yet fully understood. Understanding how microbiota influences EMT and cancer progression could lead to new treatments and therapies for various diseases, including cancer. The metabolites produced by GM lead to hypoxic conditions in the tumor microenvironment, causing cancer cells to alter their metabolic processes through key genes such as HIFs. Directly promoting the expression of molecules associated with the VM process, HIF-1α affects Snail, ZEB2, Twist, TGF-β3, and VEGF. Activating the PI3K/AKT signaling pathway by this molecular route encourages VM formation in cancer, offering a new focus for improving cancer therapies.

We contend that deliberately regulating harmful metabolites to reduce or eliminate their levels is likely an effective strategy for preventing and treating cancer. Moreover, conducting further investigations is crucial to validate the management approaches’ functional reliability, clinical efficacy, and safety. The likelihood of developing cancer varies among individuals. One contributing factor to this disparity could be the unique GM compositions found in each person, which may impact their susceptibility to cancer and their effectiveness in responding to various treatment approaches. Analyzing the roles of various GMs and their metabolic substances in angiogenesis and vascular mimicry will aid in achieving more personalized healthcare solutions. Our everyday food consumption patterns significantly shape our microbiome, which affects our health and contributes to the risk of various non-communicable diseases, such as cancer and VM. The developing microbiome sector has recognized that attaining a profound mechanistic comprehension of the dietary and microbiome influences on VM may pave the way for establishing patient-centric solutions. These solutions would equip patients and healthcare professionals with the knowledge and instruments required to prevent health issues, ease symptoms, or enhance the effectiveness of therapies. Ultimately, advancing our understanding from mere associations, correlations, and predictions to causal and mechanistic insights regarding the effects of diet and GM on VM will allow researchers to differentiate between the prevalent “passenger microbiome alterations” associated with cancer and the more significant “driver alterations” that directly influence cancer-related processes.

## Data Availability

Not applicable.

## Abbreviations

The following abbreviations are used in this manuscript:

5-ASA	5-amino salicylic acid
EGFR	Epidermal growth factor receptor
FexD	Fexaramine D
OCA	Obeticholic acid
T-βMCA	Tauro-β-muricholic acid
FXR	Farnesoid X receptor
M3R	Muscarinic acetylcholine receptor M3
LCA	Lithocholic acid
BAs	Bile acids
MFB	Metformin-butyrate
ROS	Reactive oxygen species
MAMPs	Microorganism-associated molecular patterns
TLRs	Toll-like receptors
TLRs	Through Toll-like receptors
EMT	Epithelial–mesenchymal transition
CRC	Colon cancer
LPS	Lipopolysaccharide
DCA	Deoxycholic acid
CSCs	Cancer stem-like cells
ERK	Extracellular-regulated protein kinase
MAPK	Mitogen-activated protein kinase
PI3K	Phosphatidylinositol 3 kinase
AKT	Protein kinase B
VE-PTP	Vascular endothelial protein tyrosine phosphatase
AJs	Adhesion junctions
(VE)-cadherin	Vascular endothelial
TWIST1	Twist-related protein 1
EPHA2	Ephrin type-A receptor 2
VEGF	Vascular endothelial growth factor
MMPs	Matrix metalloproteinases
TGFB	Growth factor beta 1
FMO1	Flavin-containing monooxygenase
TMA	Trimethylamine
cutC/D	Choline TMA-lyase
CntA/B	L-carnitine oxygenase
SCFAs	Short-chain fatty acids
TMAO	Trimethylamine N-oxide
GM	Gut microbiota
VM	Vasculogenic mimicry
